# Composite testing for *ante*-*mortem* diagnosis of Johne’s disease in farmed New Zealand deer: correlations between bacteriological culture, histopathology, serological reactivity and faecal shedding as determined by quantitative PCR

**DOI:** 10.1186/1746-6148-9-72

**Published:** 2013-04-10

**Authors:** Rory O’Brien, Alan Hughes, Simon Liggett, Frank Griffin

**Affiliations:** 1 Disease Research Laboratory, Department of Microbiology & Immunology, University of Otago, Dunedin, New Zealand

**Keywords:** Johne’s disease, Paratuberculosis, MAP, Quantitative PCR, ELISA, Deer, *Cervus elaphus*

## Abstract

**Background:**

In the absence of overt clinical signs of Johne’s Disease (JD), laboratory based tests have largely been limited to organism detection via faecal culture or PCR and serological tests for antibody reactivity. In this study we describe the application of quantitative faecal PCR for the detection of *Mycobacterium avium* subsp*. paratuberculosis* (MAP) in New Zealand farmed deer to quantify the bacterial load in cervine faecal samples as an adjunct to an existing serodiagnostic test (Paralisa™) tailored for JD diagnosis in deer. As ELISA has potential as a cheap, high throughput screening test for JD, an attempt was made to assess the sensitivity, specificity and positive/negative predictive (PPV/NPV) values of Paralisa™ for estimating levels of faecal shedding of MAP as a basis for JD management in deer.

**Results:**

Correlations were made between diagnostic tests (ELISA, qPCR, culture and histopathology) to establish the precision and predictive values of individual tests. The findings from this study suggest there is strong correlation between bacterial shedding, as determined by faecal qPCR, with both culture (r = 0.9325) and histopathological lesion severity scoring (r = 0.7345). Correlation between faecal shedding and ELISA reactivity in deer was weaker with values of r = 0.4325 and r = 0.4006 for Johnin and Protoplasmic antigens, respectively. At an ELISA Unit (EU) cutoff of >50 (Johnin antigen) the PPV of Paralisa™ for significant faecal shedding in deer (>10^4^ organisms/g) was moderate (0.55) while the NPV was higher (0.89). At an EU cutoff of ≥150, the PPV for shedding >10^5^ organisms/g rose to 0.88, with a corresponding NPV of 0.85.

**Conclusions:**

The evidence available from this study suggests that Paralisa™ used at a cutoff of 50EU could be used to screen deer herds for MAP infection with sequential qPCR testing used to cull all Paralisa™ positive animals that exhibit significant MAP faecal shedding.

## Background

New Zealand (NZ) is unique in that it has a population of more than 1.2 million farmed European red deer (*Cervus elaphus*), a farming practice not commonly employed internationally. Johne’s Disease (JD), caused by infection with *Mycobacterium avium* subsp*. paratuberculosis* (MAP) in deer presents as a unique syndrome and is increasingly recognised as a production limiting disease of concern to the NZ deer farming industry. In deer, JD manifests as an acute infection with progression from infection through to clinical disease or death occurring more rapidly than in cattle or sheep and with some particularly susceptible animals dying from the disease as early as eight months of age [1]. Consequently there is a need for accurate diagnostic tests for MAP infection in farmed deer, where JD may result in serious losses [2].

Deer mount a vigorous immune response to MAP infection characterised by high titres of antibody [3] and are capable of shedding large numbers of MAP organisms into the environment as the disease progresses from the paucibacillary to multibacillary state [4]. In contrast to cattle and sheep, young deer (<1 year of age) appear to be especially susceptible to challenge with the bovine strain of MAP [5] although ovine strain MAP has also been implicated in cervine JD on occasion [6]. Nonetheless, most farmed deer herds that are affected by MAP do not suffer from overt clinical losses due to JD and good management practices appear to keep disease problems at a level that is acceptable within normal farming production systems in NZ. The motivation to introduce diagnostic methods that are appropriate and effective places extreme demands on diagnostic platforms used to support control of MAP in domesticated ruminants, for a disease that is only rarely obvious as a health hazard.

As is the case for all chronic, mycobacterial diseases in humans and animals, the development and validation of sensitive and specific diagnostic methods to diagnose infection and disease is particularly challenging. Perceived imprecision of available tests reflects the biology of mycobacterial infection and the chronology of the resultant immune responses triggered within the host, coupled with the existence of closely related and antigenically very similar mycobacterial species ubiquitous within the environment. These factors dictate that, however urgently they may be needed, the development of tests that more accurately and cost effectively diagnose MAP infection or JD in domestic animals is difficult. As no existing single test ticks every box in terms of sensitivity, specificity, turnaround and cost-effectiveness, combinations of different tests are necessary to achieve optimal diagnosis. Commonly utilised *ante-mortem* diagnostic tests for JD include immunodiagnostic tests for serum antibody by ELISA or organism based tests to detect the presence of the bacterium, such as faecal culture or PCR. The specificity of serological diagnostic tests may be compromised by common antigens shared by MAP, *M. bovis* and other saprophytic environmental mycobacteria that evoke an immune response in non-diseased animals. The sensitivity of serodiagnostic tests, particularly for subclinically infected animals in the early stages of JD, is also influenced by the dynamics of antibody production and the point at which a sample is assayed due to the predominantly cellular immune responses found in the early stages of disease, limiting the predictive value of the test [7]. Performance of commercially available serodiagnostic test kits is further challenged when considering host species other than the target species for which they were developed; commercial ELISA kits may have limited or no capacity to detect antibodies from different host species such as deer [8]. While faecal culture on Herrold’s egg yolk (HEY) medium has remained the definitive test for MAP infection this requires prolonged incubation periods of up to sixteen weeks and may be compromised by overgrowth by contaminating gut microflora [9-13]. Radiometric tests (BACTEC™) have been developed as a substitute for traditional faecal culture tests and have been widely adopted as they speed up the time to detect mycobacterial growth [14].

Diagnostic tests available to deer farmers in NZ have included conventional pooled and individual faecal culture on solid and BACTEC™ medium as well as indirect ELISA and agar gel immunodiffusion, each with associated strengths and weaknesses. In addition, there is available an IgG_1_ ELISA (Paralisa™) test developed specifically for the diagnosis of MAP infection in deer through the detection of antibody to denatured Johnin (PPD-J) and native Protoplasmic (PPA) antigens; the antibody responses to both test antigens are considered in parallel to arrive at a final result [3,15]. Initial studies in deer [15,16] have suggested that subclinically affected deer produce higher levels of seroreactivity (IgG_1_) than has been shown previously in cattle [17] or sheep [18]. ELISA techniques such as the Paralisa™ lend themselves well to automation using laboratory robotics thereby reducing labour costs and turnaround time; this facet is one of the great strengths of ELISA as a screening technique as it allows whole herds or sub-populations of animals to be economically screened, quickly informing management decisions.

Because JD presents as an enteropathy and the intestine is the major site of colonisation of the causative agent, faecal samples are an obvious *ante-mortem* source material with which to attempt to diagnose MAP infection and faeces are considered to be one of the most important materials for diagnosis of JD in the live animal because it is possible to identify subclinical and clinical animals relative to the level of shedding of MAP organisms [19]. In deer, it has been reported that animals suffering from clinical JD are capable of excreting 5 × 10^6^ colony forming units of bacteria per gram of faeces [20] and that an infective dose can be as low as 10^3^ organisms [21]. Internationally, attention has increasingly turned towards rapid, nucleic acid amplification based approaches for the confirmation and quantification of MAP bacilli in faecal samples [11,12,22-28]. DNA amplification (PCR) based tests are attractive due to their low cost and high speed and have become established as a mainstream diagnostic methodology for a variety of pathogenic organisms [29]. The inclusion of fluorescent reporter dyes has allowed PCR to be used quantitatively to determine the bacterial load in individual samples allowing distinctions to be made between high, medium and low or non-shedders; this aspect of the technique could be an invaluable JD management tool for selective culling of infectious/shedding animals, to facilitate control in infected herds and accredit low-risk replacement animals for introduction into herds. A recent comparative study of qPCR [30], solid and liquid culture and ELISA for detection of MAP in cattle based on 143 samples concluded using Bayesian methodology (independent of a gold standard) that test sensitivity for culture methods and qPCR, as well as test accuracy, are comparable. Sensitivity and specificity of qPCR in this case were reported to be 0.60 and 0.97, respectively; accuracy of qPCR (0.90) was comparable to both solid (0.91) and liquid (0.93) culture leading the authors to conclude that qPCR has considerable potential to quantify MAP in faecal samples.

While there are numerous reports describing quantitation of MAP shedding in cattle and sheep, there have been no reports describing quantitation of MAP shedding in cervine faeces by PCR or any observed correlation between faecal shedding and other diagnostic parameters in deer. The current study attempts to compare diagnostic precision of ELISA, qPCR, culture and histopathological assays for MAP infection and JD in farmed deer. An inhouse qPCR assay for faecal shedding was firstly validated using proficiency panels consisting of bovine faecal samples of previously determined MAP titre and subsequently applied to a panel of 663 cervine faecal samples for which matched blood samples were available.

## Results

### Validation of qPCR and Correlation of qPCR with culture techniques

**Figure 1:**
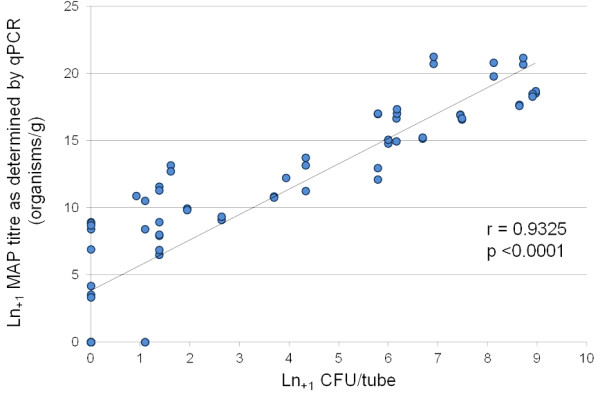
**Correlation between MAP titre as determined by qPCR and faecal culture on HEYM.** Correlation of faecal qPCR data against HEYM solid culture based on cumulative NVSL JD Proficiency Panel Samples for 2008, 2009 and 2010; *n* = 72 (of a total of 78 individual faecal samples, two did not have associated colony counts and four were disregarded for official grading purposes). Spearman Correlation = 0.9325; p <0.0001.

In order to evaluate the precision of an inhouse faecal qPCR of cervine samples against conventional culture-based MAP quantification methodologies a proficiency panel of bovine faecal samples of known infection status and faecal culture titre, administered through the US National Veterinary Services Laboratory (NVSL), Ames, Iowa as part of an ongoing JD proficiency testing panel for diagnostic laboratories was used [31]. The NVSL JD Proficiency Panels are distributed annually to diagnostic and research laboratories in the US and internationally and are used to accredit testing services for JD diagnostic testing in the US. Each panel consists of 26 blinded-duplicate (bovine) faecal samples, with one sample identified as a positive control, and includes designated critical-pass positive and negative samples along with samples spiked with mycobacterial species other than MAP. Positive samples were collected from naturally infected cows and negative samples from individual cattle in non-infected herds. Faecal samples comprising cumulative proficiency panels from 2008 – 2010 were assessed (*n* = 78). The qPCR assay identified 26/26 (100%) of the isolates correctly from the 2008 panel, which included one sample spiked with *M. avium,* which was correctly identified as MAP negative. Using the 2009 panel one of the duplicates for animal 19, a very low shedder, was misclassified as negative by qPCR when solid culture had returned a result of 2 CFU/tube although the duplicate sample from this animal was correctly identified. Overall for the 2009 panel, 24/25 (96%) of the isolates were correctly identified. The 2009 panel also included one sample spiked with *M. fortuitum* which was correctly identified as MAP negative. The 2010 proficiency panel included four low shedding samples which were not included for official grading purposes by the panel administrators as shedding levels were so low that <70% were identified as positive by participating laboratories, leaving 21 valid samples; of these, 21/21 (100%) were correctly identified. The retrospective assessment of the’08,’09 and’10 NVSL panels indicated that qPCR correctly identified 72/73 (99%) of the valid diagnostic specimens. The observed correlation in organism quantitation between faecal qPCR and MAP culture on HEY solid medium (Spearman r = 0.9325; p <0.0001) based on the NVSL JD Proficiency Panels is illustrated in Figure 1 and the combined results for the three proficiency panels are reported in Additional file 1: Table S1; these data confirmed that the inhouse qPCR methodology utilised for quantitation of MAP in cervine faeces was returning meaningful results. While the qPCR validation results were based on bovine faecal samples, no significant differences in the analysis of cervine faeces were anticipated. The proficiency panel dataset also allowed sensitivity and specificity values to be calculated for the qPCR assay at different shedding thresholds taking culture on HEY solid medium as a gold standard; these data are presented in Table 1.

**Table 1:** Sensitivity and specificity values for qPCR calculated using HEYM culture as a gold standard

	**Shedding threshold for qPCR test positivity (organisms/g)**						
	**10**^**-1**^	**10**^**1**^	**10**^**2**^	**10**^**3**^	**10**^**4**^	**10**^**5**^	**10**^**6**^
Sens.	0.91	0.91	0.91	0.85	0.68	0.52	0.37
Spec.	0.73	0.73	0.83	0.89	1.00	1.00	1.00
PPV	0.91	0.91	0.94	0.95	1.00	1.00	1.00
NPV	0.74	0.74	0.75	0.66	0.52	0.42	0.35

Sensitivity and specificity values for qPCR calculated for different shedding states using HEY solid medium as a gold standard based on NVSL proficiency panel culture results.

### Correlation between qPCR and Histopathological Severity Score

**Figure 2:**
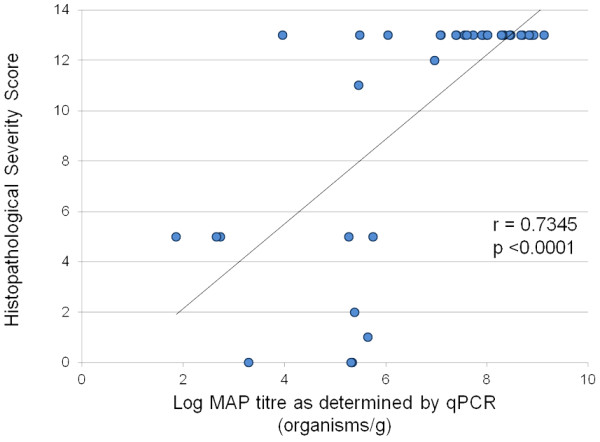
**Correlation between MAP titre as determined by qPCR and Histopathological Lesion Severity Score.** Correlation of faecal MAP titre as determined by qPCR and Histopathological Lesion Severity Score; *n* = 40, Spearman Correlation r = −0.7345; p <0.0001.

A recently described histopathological severity scoring system for the objective description of JD pathology in deer [4] was used to compare active disease as defined by histopathology scores to faecal shedding as determined by qPCR. This scoring system ranks JD histopathological severity on a numerical scale from 0 (normal histology with no lesions or acid-fast organisms visible) through low to moderate grade tissue damage (with increasing score values reflecting increasing numbers of granulomas, submucosal lesions and bluntening of *villi*) up to a maximum score of 13 (encompassing large areas of granulomatous lesions (including caseated calcified lesions) in intestine or lymph node, submucosal lesions, marked bluntening of *villi* and granulomas in lymph node capsule and/or serosa/mesentery). Examination of a small dataset of samples (*n* = 40) for which matched histopathological severity scoring and faecal qPCR data were both available, revealed a significant correlation (Spearman r = 0.7345; p <0.0001), with higher levels of shedding associated with samples obtained from animals with the most severe histopathological lesion severity scores and reduced shedding in samples of lesser disease severity (Figure 2). Within this small dataset there were two samples with histological severity scores of 0 which estimated faecal shedding of >10^3^ and >10^5^ organisms/g respectively, which may be due to sampling error limitations associated with tissue sampling. However, of the 28/40 samples which recorded the maximum histopathological severity score of 13, 26/28 (93%) were determined to be shedding >10^6^ organisms/g. These data suggest that significant faecal shedding of MAP is a reasonable indicator of clinical JD as determined by histopathological analysis.

### Correlation of qPCR with ELISA using cervine samples

**Figure 3:**
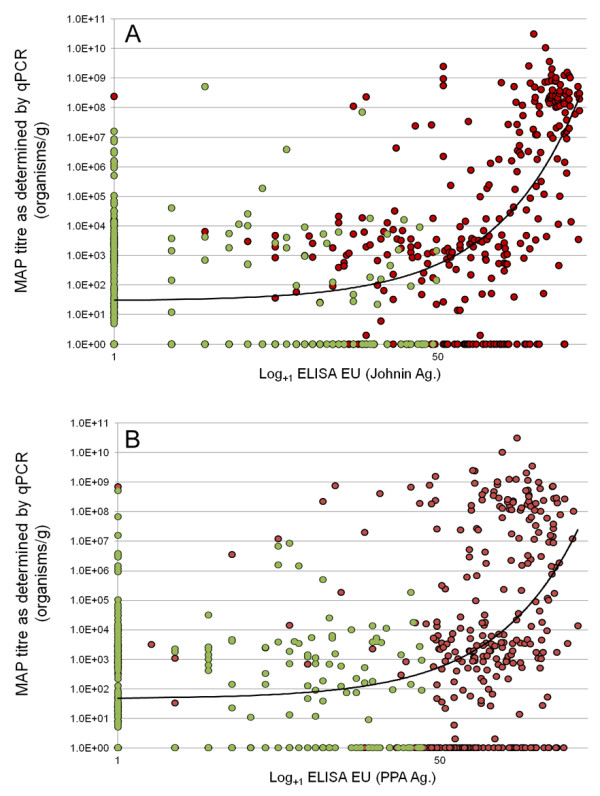
**Correlation between ELISA reactivity and MAP titre as determined by qPCR.** Panel **A**; Correlation of faecal MAP titre as enumerated by qPCR vs ELISA (Paralisa™) reactivity of matched cervine blood samples (Johnin antigen); Spearman correlation r = 0.4325; p <0.0001. Panel **B**; Correlation of faecal MAP titre as enumerated by qPCR vs ELISA (Paralisa™) reactivity of matched blood samples (Protoplasmic antigen); Spearman correlation r = 0.4006; p < 0.0001. *n* = 663. Red circle = Overall Paralisa™ result = positive; Light green circle = Overall Paralisa™ result = negative.

While MAP qPCR and the Paralisa™ (ELISA) assay distinct biological parameters (organism enumeration vs. immunological response), it seemed reasonable to assume that MAP shedding would correlate with disease severity and also serological reactivity. As the disease state cannot be quantified in *ante-mortem* samples, we examined matched data from 663 cervine blood samples submitted for routine JD testing by Paralisa™ (PPD-J) for which faecal samples had also been obtained and submitted at the time of blood sampling. The correlation between qPCR and ELISA was not as strong as that observed for qPCR and culture, although a significant correlation (Spearman r = 0.4325; p <0.0001) was observed, suggesting that with strongly positive Paralisa™ results, there was a greater chance that the animal was shedding high numbers of organisms in its faeces. Conversely, low antibody reactivity corresponded with lower incidence of MAP faecal shedding. These data are illustrated in Figure 3.

In light of the fact that neither ELISA nor qPCR provide binary (positive/negative) but rather scalar data and rely on cutpoints to be assigned to differentiate positive from negative samples, any value for sensitivity or specificity for these tests becomes cutpoint dependant. By considering multiple cutpoint thresholds for positivity it was possible to calculate values for sensitivity, specificity and for positive and negative predictive values for serological reactivity for a range of positive outcomes and for a range of shedding states (Table 2). In this dataset the negative predictive value of Paralisa™ for moderate bacterial shedding (>10^4^ organisms/g was 0.90. With higher levels of faecal shedding (≥10^6^ organisms/g) the NPV rose to 0.97 and at the highest shedding levels detected (≥10^8^ organisms/g) NPV was 1.00. Few (16/663; 2.4%) Paralisa™ negative animals (PPD-J; <50EU) were found to shed moderate or high (>10^5^ organisms/g) numbers of MAP in their faeces. By contrast, the positive predictive value (PPV) of Paralisa™ for faecal shedding (≥10^4^ organisms/g) was 0.55, suggesting that a Paralisa™ positive result with a cutoff value for positivity of ≥50EU is not strongly predictive for shedding. At ≥150EU, however, PPV for Paralisa™ rose to 0.88 with a corresponding NPV of 0.85. In a recent review of JD in cattle and other susceptible species Sweeney *et al.* (2012) urged clinicians not to accept simple dichotomous (positive/negative) ELISA results but rather to interpret quantitative results (ELISA Optical density (OD) or Standard/Positive (S/P) ratio) which correlate well with the likelihood of (and degree of) faecal shedding of MAP [32,33]; this viewpoint would appear to be strongly supported when considering test results in deer also.

**Table 2:** **Sensitivity and specificity values for Paralisa**™ **calculated using faecal qPCR as a gold standard**

		**Shedding Threshold for Positivity (organisms/g)**						
		**≥10**^**2**^	**≥10**^**3**^	**≥10**^**4**^	**≥10**^**5**^	**≥10**^**6**^	**≥10**^**7**^	**≥10**^**8**^
Paralisa Overall	Sens.	0.62	0.67	0.78	0.87	0.89	0.96	0.98
	Spec.	0.70	0.68	0.63	0.62	0.61	0.60	0.58
	PPV	0.70	0.61	0.41	0.35	0.31	0.26	0.20
	NPV	0.62	0.74	0.90	0.95	0.97	0.99	1.00
PPD-J EU >50	Sens.	0.46	0.52	0.70	0.82	0.84	0.91	0.94
	Spec.	0.85	0.84	0.81	0.81	0.79	0.78	0.75
	PPV	0.78	0.71	0.55	0.50	0.44	0.37	0.28
	NPV	0.58	0.70	0.89	0.95	0.96	0.98	0.99
PPD-J EU >100	Sens.	0.34	0.40	0.62	0.74	0.78	0.86	0.89
	Spec.	0.94	0.93	0.93	0.92	0.91	0.89	0.86
	PPV	0.87	0.82	0.73	0.67	0.62	0.53	0.40
	NPV	0.55	0.68	0.88	0.94	0.95	0.98	0.99
PPD-J EU >150	Sens.	0.24	0.29	0.48	0.60	0.64	0.75	0.81
	Spec.	0.98	0.98	0.98	0.97	0.96	0.96	0.93
	PPV	0.93	0.92	0.88	0.83	0.78	0.71	0.56
	NPV	0.53	0.65	0.85	0.91	0.93	0.96	0.98

Sensitivity (Sens.), specificity (Spec.), positive and negative predictive values (PPV and NPV, respectively) for Paralisa^TM^ to predict MAP shedding in deer as determined by qPCR. ‘Paralisa Overall’ indicates the final result when Johnin and Protoplasmic Antigen responses are interpreted in parallel; EU, ELISA Units.

Instances where Paralisa™ positive samples exhibited low or no shedding in matched faecal samples may be explained through intermittency of shedding, sampling error or the stage of the MAP infection cycle at which samples were tested, particularly in subclinical individuals. When considering the data provided in Table 2, however, it should be noted that these data do not provide a true estimate of test specificity, as most of the data was derived from herds where MAP infection was present; a more accurate estimate of test specificity would become evident if the dataset for specificity estimates were derived using herds free from MAP infection.

## Discussion

Diagnosis of JD in the live animal is challenging and the prompt identification of animals shedding significant numbers of MAP bacilli would be a valuable management tool. Earlier published data [3] showed an estimated sensitivity of 0.77 for a customized ELISA (Paralisa™) test to diagnose MAP infection in deer. While this test has been used successfully to control infection in a test and cull programme [15] there was concern that this approach could result in wastage of valuable stud stock as false positive reactors. The current study provides evidence that faecal qPCR could be used as a sequential ancillary to the Paralisa™. Because qPCR is comparatively labour intensive and expensive it is not practical for use on individual animals as a whole herd screening test and it has been used as a research tool rather than as a frontline diagnostic test in NZ. The selective use of faecal qPCR as a sequential ancillary test for whole herd tested ELISA positive animals either as individual or pooled tests, brings such testing into the realm of practicable application.

Considering the ubiquitous nature of MAP within infected herds the intended purpose of the faecal qPCR test was not to diagnose the presence of MAP *per se*, but rather to diagnose animals shedding a minimal threshold of bacilli to justify selective culling, or to confirm the JD status in high-value animals using ancillary testing. The value of faecal qPCR testing relates to the speed with which it can identify infectious or affected high shedder animals that pose a risk for environmental spread of infection. As qPCR becomes increasingly accepted and adopted as a routine JD diagnostic methodology, it will become necessary to determine what level of MAP shedding may be indicative of passive transfer of MAP arising from a contaminated environment as opposed to an infectious, affected or diseased state. The detection of very low/trace numbers of MAP organisms in ruminant faeces may not be informative or useful until we have a clear understanding as to what may constitute normal bacterial shedding or pass-through of an environmental contaminant through the intestine of a subclinically infected, yet unaffected host. Ultimately, the test parameters used must be dictated by the purpose for which the test is applied whether it be whole herd screening, testing of replacements prior to bringing animals onto a property or selection of animals for culling. The prevalence of MAP infection and degree of environmental contamination must also be taken into account and no single set of parameters will satisfy universal requirements. Nonetheless, tests that can efficiently detect animals shedding high numbers in their faeces are vital for an effective JD management programme and the early identification and prompt removal of such shedders is generally accepted as essential.

While we do not yet understand what level of shedding may mark a transition from exposure through to subclinical infection and development of clinical disease, longitudinal testing of larger number of animals using sequential diagnostic tests will identify threshold values that correlate with these states. While it is known that faecal shedding of MAP in deer can be intermittent, especially in subclinical individuals, it is likely that significantly affected animals will remain as persistent and consistent high level shedders. While terminal JD results in scouring and wastage of affected deer, a significant proportion of high shedding animals may show no evidence of scouring, indeed some of the highest MAP titres detected as part of this study were recovered from well formed, solid faecal pellets as opposed to liquefied faeces. It has similarly been reported that cattle showing no clinical signs of JD or scouring may be capable of shedding >10^6^ CFU of MAP per gram of faeces [24].

Polymerase Chain Reaction based tests offer potential for continued development, particularly in regard to sample preparation, to further improve upon sensitivity, automation, throughput and to achieve improved cost-effectiveness. In particular there is scope for semi-automation of DNA extraction through the use of improving extraction technologies incorporating laboratory robotics; the setup and execution of the PCR itself is similarly amenable to automation. The development of improved PCR is ongoing in research laboratories internationally and increasing numbers of commercial suppliers are developing PCR tests for MAP in kit form, all of which will continue to advance PCR as a valid alternative to conventional culture methodologies. Culture based approaches, by comparison, are less likely to be significantly improved upon due to the inherent limitation resulting from the slow growth rate of this organism in culture and difficulties associated with its early detection and quantitation.

## Conclusions

Data obtained with qPCR compares favourably with culture in terms of sensitivity, cost and speed but will remain more expensive than ELISA for MAP diagnosis. While MAP culture is more sensitive in detecting animals shedding few MAP organisms, it is questionable whether sensitivity and precision to distinguish the presence of MAP (the organism) or a host affected by JD is most relevant. The results reported here suggest that a rapid qPCR assay for MAP in deer faeces could provide another tool in the JD management toolkit. Current data on qPCR in farmed deer, used in sequence with Paralisa™ could become a practical strategy to identify and cull animals shedding significant numbers of MAP*.* Use of this technology could limit the spread of MAP infection, minimise production/reproduction losses from infection, and provide increased food safety and market assurance.

## Methods

### ELISA

The methodology outlined in this paper was based on previously published parameters for ELISA immunoassays (Paralisa™) used to diagnose immune reactions in deer to MAP infection [3]. This serological test quantifies antibody responses to two distinct protein antigens in parallel, a denatured antigen in the form of Purified Protein Derivative – Johnin (PPD-J) and a native protein in the form of Protoplasmic Antigen (PPA); final test results are arrived at by considering both test antigens in parallel. Deer disease severity was based on histopathology severity scores, using necropsy specimens obtained from natural and experimental infection studies in deer involving virulent MAP [4].

### qPCR

Levels of MAP shedding in cervine faecal samples were determined using an inhouse qPCR assay. Descriptions of a number of qPCR based assays for quantitation of MAP have appeared in the literature in recent years and quantitation of MAP bacilli in faecal material using qPCR, competently employed, is now considered routine and to be on a par with faecal culture in terms of diagnostic performance [32,34]. In brief, the current qPCR assay utilised F57, a single-copy, species-specific sequence diagnostic target [35,36], using hydrolysis probe based chemistry. Every assay included an internal amplification control molecule labelled with a second reporter dye co-amplified alongside the diagnostic target in a duplex format to exclude false negative reactions and to disclose any inhibition of the reaction; appropriate biological and technical replicates and necessary positive and negative controls were also included. Quantitation of MAP titre was accomplished by preparing a standard curve using serial log dilutions of MAP bacilli. DNA from cervine faecal samples (3g) was homogenised and total DNA extracted and purified using a combination of physical and chemical methods; final cleanup and removal of any coextracted inhibitors was achieved using a commercial faecal DNA extraction kit (Zymo Research Corp., CA, USA). Both diagnostic target and internal control amplification assays each displayed amplification efficiencies of 100%.

## Abbreviations

MAP: *Mycobacterium avium* subsp*. paratuberculosis*; JD: Johne’s disease; NZ: New Zealand; ELISA: Enzyme linked immunosorbent assay; EU: ELISA unit; PPD-J: Purified protein derivative – Johnin; PPA: Protoplasmic antigen; PCR: Polymerase chain reaction; qPCR: Quantitative polymerase chain reaction; PPV: Positive predictive value; NPV: Negative predictive value; HEYM: Herrold’s egg yolk medium; NVSL: National veterinary services laboratory.

## Competing interests

The authors declare that they have no competing interests.

## Authors’ contributions

ROB, SL and FG conceived the study; ROB, AH and SL performed the laboratory work; ROB and FG prepared and drafted the manuscript; all authors read and approved the final manuscript.

## Supplementary Material

Additional file 1: Table S1 Validation of faecal qPCR against cumulative USDA proficiency panels from 2008 – 2010. Click here for file
