# Supplementary material for: Composite testing for ante-mortem diagnosis of Johne’s disease in farmed New Zealand deer: correlations between bacteriological culture, histopathology, serological reactivity and faecal shedding as determined by quantitative PCR
Source: BMC Vet Res. 2013 Apr 10;9:72. doi: 10.1186/1746-6148-9-72 (PMC3639118; doi:10.1186/1746-6148-9-72)
Supplement: Additional file 1: Table S1 — Validation of faecal qPCR against cumulative USDA proficiency panels from 2008 – 2010. [file 1746-6148-9-72-S1.doc]

**Additional file 1: Table S1** **Validation of faecal qPCR against cumulative USDA proficiency panels from 2008 – 2010**

|  | **Animal** | **Blinded Duplicate Vial** | **Mean IAC Ct** | **qPCR Result** | **qPCR Shedding** | **Estimated Shedding (organisms/g approx.)** | **Result** | **Mean CFU/tube^1^** | **NVSL Infection Status** |
| --- | --- | --- | --- | --- | --- | --- | --- | --- | --- |
| 2008 NVSL Proficiency Panel | 1 | A | 19.4 | Neg | Nil | Not detected | Correct | 0 | Non-infected |
|  |  | B | 19.3 | Neg | Nil | Not detected | Correct |  |  |
|  | 2 | A | 19.3 | Neg | Nil | Not detected | Correct | 0 | Non-infected |
|  |  | B | 19.2 | Neg | Nil | Not detected | Correct |  |  |
|  | 3 | A | 19.3 | Neg | Nil | Not detected | Correct | 0 | Non-infected |
|  |  | B | 19.3 | Neg | Nil | Not detected | Correct |  |  |
|  | 4 | -^3^ | 19.2 | Neg | Nil | Not detected | Correct | 0 | Non-infected |
|  | 5 | A | 19.4 | Neg | Nil | Not detected | Correct | 0 | Non-infected |
|  |  | B | 19.4 | Neg | Nil | Not detected | Correct |  |  |
|  | 6 | A | 19.4 | Pos | Low | 6.7 × 10^2^ | Correct | 3 | Infected |
|  |  | B | 19.4 | Pos | Low | 3.1 × 10^3^ | Correct |  |  |
|  |  | C | 19.4 | Pos | Low | 9.5 × 10^2^ | Correct |  |  |
|  | 7 | A | 19.4 | Pos | Low | 2.2 × 10^4^ | Correct | 6 | Infected |
|  |  | B | 19.3 | Pos | Low | 1.9 × 10^4^ | Correct |  |  |
|  | 8 | A | 19.4 | Pos | Low | 9.0 × 10^3^ | Correct | 13 | Infected |
|  |  | B | 19.3 | Pos | Low | 1.1 × 10^4^ | Correct |  |  |
|  | 9 | A | 19.2 | Pos | Mod | 5.2 × 10^4^ | Correct | 39 | Infected |
|  |  | B | 19.1 | Pos | Mod | 4.7 × 10^4^ | Correct |  |  |
|  | 10 | A | 19.3 | Pos | High | 2.6 × 10^7^ | Correct | 480 | Infected |
|  |  | B | 19.3 | Pos | High | 3.5 × 10^7^ | Correct |  |  |
|  | 11 | A | 19.4 | Pos | High | 2.3 × 10^7^ | Correct | 1725 | Infected |
|  |  | B | 19.3 | Pos | High | 2.2 × 10^7^ | Correct |  |  |
|  | 12 | A | 19.3 | Pos | High | 9.6 × 10^8^ | Correct | 6075 | Infected |
|  |  | B^2^ | 19.4 | Pos | Very High | 1.6 × 10^9^ | Correct |  |  |
|  | 13 | A | 19.3 | Pos | High | 1.1 × 10^8^ | Correct | 7850 | Infected |
|  |  | B | 19.4 | Pos | High | 1.4 × 10^8^ | Correct |  |  |
| 2009 NVSL Proficiency Panel | 14 | A | 18.8 | Neg | Nil | Not detected | Correct | 0 | Non-infected |
|  |  | B | 18.6 | Neg | Nil | Not detected | Correct |  |  |
|  | 15 | A | 18.7 | Neg | Nil | Not detected | Correct | 0 | Non-infected |
|  |  | B | 18.7 | Neg | Nil | Not detected | Correct |  |  |
|  | 16 | A | 18.7 | Neg | Nil | Not detected | Correct | 0 | Non-infected |
|  |  | B | 18.8 | Neg | Nil | Not detected | Correct |  |  |
|  | 17 | -^4^ | 18.8 | Neg | Nil | Not detected | Correct | 0 | Non-infected |
|  | 18 | - | 18.7 | Pos | Low | 5.3 × 10^4^ | Correct | 1.5 | Infected |
|  | 19 | A | 18.6 | Neg | Nil | 4.5 × 10^3^ | *Incorrect* | 2 | Infected |
|  |  | B | 19.0 | Pos | Low | 3.7 × 10^4^ | Correct |  |  |
|  | 20 | A | 18.6 | Pos | Low | 1.1 × 10^5^ | Correct | 3 | Infected |
|  |  | B | 18.7 | Pos | Low | 8.2 × 10^4^ | Correct |  |  |
|  | 21 | A | 18.7 | Pos | Low | 5.2 × 10^5^ | Correct | 4 | Infected |
|  |  | B | 18.7 | Pos | Low | 3.4 × 10^5^ | Correct |  |  |
|  | 22 | A | 18.7 | Pos | Mod | 2.4 × 10^7^ | Correct | 325 | Infected |
|  |  | B | 18.7 | Pos | Mod | 2.5 × 10^7^ | Correct |  |  |
|  | 23 | A | 18.8 | Pos | High | 1.0 × 10^9^ | Correct | 1000 | Infected |
|  |  | B | 18.6 | Pos | Very High | 1.8 × 10^9^ | Correct |  |  |
|  | 24 | A | 18.8 | Pos | Mod | 1.6 × 10^7^ | Correct | 1775 | Infected |
|  |  | B | 18.7 | Pos | Mod | 1.8 × 10^7^ | Correct |  |  |
|  | 25 | A | 18.7 | Pos | High | 1.1 × 10^9^ | Correct | 3350 | Infected |
|  |  | B | 18.7 | Pos | High | 4.0 × 10^8^ | Correct |  |  |
|  | 26 | A | 18.8 | Pos | High | 4.9 × 10^7^ | Correct | 5625 | Infected |
|  |  | B^2^ | 18.5 | Pos | High | 4.5 × 10^7^ | Correct |  |  |
|  | 27 | A | 18.9 | Pos | High | 1.1 × 10^8^ | Correct | 7375 | Infected |
|  |  | B | 18.9 | Pos | High | 8.7 × 10^7^ | Correct |  |  |
| 2010 NVSL Proficiency Panel | 28 | A | 19.2 | Neg | Nil | Not detected | Correct | 0 | Neg |
|  |  | B | 19.1 | Neg | Nil | Not detected | Correct |  |  |
|  | 29 | A | 19.0 | Neg | Nil | Not detected | Correct | 0 | Neg |
|  |  | B | 19.0 | Neg | Nil | Not detected | Correct |  |  |
|  | 30 | A | 19.1 | Neg | Nil | Not detected | Correct | 0 | Neg |
|  |  | B | 19.0 | Neg | Nil | Not detected | Correct |  |  |
|  | 31 | A | 19.0 | Neg | Nil | Not detected | *Incorrect^6^* | 2 | Very Low |
|  |  | B | 19.1 | Neg | Nil | Not detected | *Incorrect^6^* |  |  |
|  | 32 | A | 19.0 | Neg | Nil | Not detected | *Incorrect^6^* | 2 | Very Low |
|  |  | B | 19.3 | Neg | Nil | Not detected | *Incorrect^6^* |  |  |
|  | 33 | A | 19.0 | Pos | Sus | 2.8 × 10^3^ | Correct | 3 | Low |
|  |  | B | 18.9 | Pos | Sus | 7.7 × 10^3^ | Correct |  |  |
|  | 34 | - | 19.1 | Pos | Low | 2.0 × 10^5^ | Correct | 50 | High |
|  | 35 | - | 19.1 | Pos | Low | 7.7 × 10^4^ | Correct | 75 | High |
|  | 36 | A | 19.0 | Pos | Mod | 4.3 × 10^5^ | Correct | 325 | High |
|  |  | B | 19.1 | Pos | Low | 1.8 × 10^5^ | Correct |  |  |
|  | 37 | A^5^ | 19.0 | Pos | Low | 1.1 × 10^5^ | Correct | Undet. | High |
|  |  | B^5^ | 19.1 | Pos | Low | 1.3 × 10^5^ | Correct |  |  |
|  | 38 | A | 19.0 | Pos | Mod | 3.6 × 10^6^ | Correct | 400 | High |
|  |  | B | 19.1 | Pos | Mod | 2.7 × 10^6^ | Correct |  |  |
|  | 39 | A | 19.0 | Pos | Mod | 3.2 × 10^6^ | Correct | 475 | High |
|  |  | B | 18.9 | Pos | High | 1.8 × 10^7^ | Correct |  |  |
|  | 40 | A | 19.0 | Pos | Mod | 4.2 × 10^6^ | Correct | 800 | High |
|  |  | B | 19.0 | Pos | Mod | 3.8 × 10^6^ | Correct |  |  |
|  | 41 | A | 19.0 | Pos | Mod | 9.3 × 10^5^ | Correct | 75 | High |
|  |  | B | 19.0 | Pos | Mod | 5.4 × 10^5^ | Correct |  |  |

^1^Colony counts were determined by NVSL using HEY media, averaging results from 3 cultures for each cow. For high shedders the inoculum was diluted 10^-x^ until colony counts were under 100 per tube; ^2^Designated Positive Control Samples; ^3^Sample spiked with *Mycobacterium avium*; ^4^Sample spiked with *Mycobacterium fortuitum*; ^5^Bison Strain *M. ptb*, colony counts were not reported for this isolate by NVSL; ^6^Extremely low shedders in the 2010 panel omitted from official grading as <70% of samples were identified as positive by participating laboratories. IAC Ct, Internal Amplification Control threshold cycle number.
